# Early and late left ventricular effects of breast cancer chemotherapy: a prospective multi-centre study using advanced cardiac imaging

**DOI:** 10.1186/1532-429X-15-S1-P142

**Published:** 2013-01-30

**Authors:** Suchi Grover, Carmine DePasquale, Govindarajan Srinivasan, Darryl P Leong, Adhiraj Chakrabarty, Kerry A Cheong, Rohit Joshi, Amy Penhall, Majo Joseph, Bogda Koczwara, Joseph Selvanayagam

**Affiliations:** 1 Flinders Medical Centre, Bedford Park, SA, Australia; 2 Cardiology, Flinders University, Adelaide, SA, Australia; 3 Oncology, Flinders Medical Centre, Adelaide, SA, Australia; 4 Adelaide Cancer Centre, Adelaide, SA, Australia; 5 Oncology Department, Lyell McEwin Hospital, Adelaide, SA, Australia

## Background

Myocardial dysfunction is a recognized toxicity of anthracycline (A) and herceptin (H) chemotherapy. Whilst there is much current focus on the incidence and magnitude of myocardial dysfunction following the A/H regimen, whether these changes are mediated by reversible or irreversible myocardial injury remains unknown . We sought to determine rates of persistent LV dysfunction at 12 months (as defined by left ventricular ejection fraction (LVEF) decrease by 10% or below lower limits of normal) following A/H and explore the mechanism of myocardial dysfunction using advance cardiac imaging.

## Methods

Thirty six chemonaive patients (26 with A, 10 with H) with breast cancer, underwent serial CMR imaging (for LV volumes/EF, myocardial edema and necrosis) and advanced echocardiography for global longitudinal strain (GLS) in addition to standard measurements of LV diastolic function. All tests were conducted at baseline, 1, 3 and 12 months (21 patients). Six normal volunteers underwent the same CMR protocol. For edema imaging, short-TI inversion recovery was used in 3 short-axis views of the LV. Myocardial edema was assessed using Lake Louis criteria [1].

## Results

In the study patients, the mean CMR LV end-systolic volume index (LVESVI) increased from baseline of 17.4 ± 6.1 to 26.4 ± 9.6; p value <0.001 at 12 months (Table 1). LVEF decreased from 72.4 ± 6.4 to 64.8 ± 6.4 at 12 months; p value < 0.001. Significant functional changes were seen as early as 1 and 3 months (table 1). Fifty % (9/21) of patients exhibited persistent LV myocardial systolic dysfunction at 12 months. GLS showed no significant change at 1 month, however decreased in absolute magnitude from -21.2 ± 3% baseline to -19.2 ± 1.8% (p ≤ 0.001) at 3 months. Although there was no difference between baseline values of T2 ratio between normal volunteers and study patients, 52% (17/33) of patients had an abnormal T2 signal as specified by Lake Louis criteria in one or more short axis slices 3 months post chemotherapy. One patient developed new late gadolinium hyperenhancement suggestive of focal myocardial necrosis/fibrosis in the trastuzumab group at 3 months following therapy.

**Table 1:** Functional changes following chemotherapy

	LVEDVI	LVESVI	LVEF	LVMM	RVEDVI	RVESVI	RVEF
Baseline (n=33)	63±12	17±6	72±6	48±9	66±14	25±8	63±8
1 month (n=33)	64±11	20±6**	69±6**	50±9	69±15	27±8*	60±6*
3 months (n=33)	65±12	21±7**	68±6**	48±9	67±13	28±7**	58±6**
12 months (n=21)	73±14**	26±10**	65±6**	40±6	71±11*	35±7**	51±9*

*p <0.05 **p<0.001

## Conclusions

Our data suggest that the subtle functional changes in the LV myocardium early post breast cancer chemotherapy persist into 12 months in a significant number of patients. These changes are likely mediated by myocardial inflammation, and despite absence of irreversible myocardial injury, continue to 12 months post therapy. These findings warrant caution and suggest ongoing regular follow-up of patients following chemotherapy is required.

## Funding

nil
